# Sixth Registration Report of South Carolina

**Published:** 1861-01

**Authors:** 


SIXTH REGISTRATION REPORT OF SOUTH CAROLINA; containing the report of Births, Marriages, and Deaths, in the State for the year ending Dec. 31st, 1859. By ROBERT W. GIBBS, Jr., M. D. Register.

This is a volume of 116 pages, mostly occupied with carefully prepared tubular statements in reference to the three topics above named. The report is enhanced by giving the statistics of the two races (White and Black) separately. Dr. Gibbs has performed his task well; and we hope the time is not far distant when similar reports will be made annually in every State of North-America.

